# Elevated concentrations of Neu5Ac and Neu5,9Ac_2_ in human plasma: potential biomarkers of cardiovascular disease

**DOI:** 10.1007/s10719-023-10138-3

**Published:** 2023-11-22

**Authors:** Jack Cheeseman, Concepcion Badia, Georgia Elgood-Hunt, Richard A. Gardner, Duong N. Trinh, Marco P. Monopoli, Gunter Kuhnle, Daniel I.R. Spencer, Helen M.I. Osborn

**Affiliations:** 1 School of Pharmacy, University of Reading, Whiteknights, Reading, RG6 6AD UK; 2 Ludger Ltd, Culham Science Centre, Abingdon, OX14 3EB UK; 3 Department of Chemistry, Royal College of Surgeons in Ireland (RCSI), Dublin 2, Dublin, D02 YN77 Ireland; 4 Department of Food and Nutritional Sciences, University of Reading, Whiteknights, Reading, RG6 6AH UK; 5 Department of Pharmaceutics and Pharmaceutical Technology, University of Medicine and Pharmacy, Vietnam National University, Hanoi, Vietnam

**Keywords:** Sialic acid, Biomarker, Cardiovascular Disease, Inflammation, ROC analysis

## Abstract

Cardiovascular disease (CVD) is a group of health conditions affecting the heart and vascular system with very high prevalence and mortality rates. The presence of CVD is characterised by high levels of inflammation which have previously been associated with increased plasma concentrations of *N*-acetyl neuraminic acid (Neu5Ac). While Neu5Ac has been studied in the context of CVD, Neu5,9Ac_2_ has not, despite being the second most abundant sialic acid in human plasma. A small-scale pilot study of thirty plasma samples from patients with diagnosed CVD, and thirty age and sex-matched healthy controls, was designed to gain insight into sialic acids as biomarkers for CVD and potential future areas of study. Each sample was assayed for Neu5Ac and Neu5,9Ac_2_ concentrations. Mean Neu5Ac and Neu5,9Ac_2_ concentrations were significantly elevated in patients with CVD compared to healthy controls (Neu5Ac: P < 0.001; Neu5,9Ac_2_: P < 0.04). Receiver operator curve (ROC) analysis indicated that both Neu5Ac and Neu5,9Ac_2_ have reasonable predictive power for the presence of CVD (Neu5Ac AUC: 0.86; Neu5,9Ac_2_ AUC: 0.71). However, while Neu5Ac had both good sensitivity (0.82) and specificity (0.81), Neu5,9Ac_2_ had equivalent specificity (0.81) but very poor sensitivity (0.44). A combination marker of Neu5Ac + Neu5,9Ac_2_ showed improvement over Neu5Ac alone in terms of predictive power (AUC: 0.93), sensitivity (0.87), and specificity (0.90). Comparison to a known inflammatory marker, high sensitivity c-reactive protein (hs-CRP: P-value: NS, ROC:0.50) was carried out, showing that both Neu5Ac and Neu5,9Ac_2_ outperformed this marker. Further to this, hs-CRP values were combined with the three different sialic acid markers to determine any effect on the AUC values. A slight improvement in AUC was noted for each of the combinations, with Neu5Ac + Neu5,9Ac_2_ + hs-CRP giving the best AUC of 0.97 overall. Thus, Neu5Ac would appear to offer good potential as a predictive marker for the presence of CVD, which the addition of Neu5,9Ac_2_ predictive power improves, with further improvement seen by the addition of hs-CRP.

**Supplementary Information:**

The online version contains supplementary material available at 10.1007/s10719-023-10138-3.

## Introduction

CVD is one of the leading causes of death and morbidity worldwide accounting for 34% (20.2 million) of deaths in 2022 with an estimated 500 million active cases [1]. CVD is associated with increased levels of inflammation, especially in the vascular endothelium. This inflammation may contribute to the progression of CVD and cause myocardial and vascular damage [2]. Accurate, earlier diagnosis of CVD allows for swifter intervention and treatment which may help lower the global healthcare burden and mortality rates associated with CVD. Biomarkers play a key role in this. Some markers for inflammation such as high sensitivity c-reactive protein (hs-CRP) have previously been studied in the context of CVD [3]. It has been shown, however, that hs-CRP may underestimate inflammation and therefore have lower predictive power than that required for diagnosis or prediction of CVD onset [4]. This highlights the need for biomarkers of CVD that have high predictive power and the ability to discriminate between CVD cases and healthy controls.

**Fig. 1:**
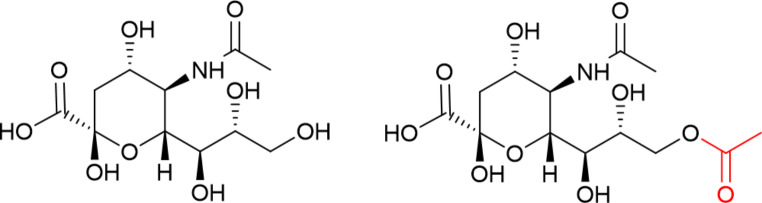
NeuAc (left) and Neu5,9Ac_2_ (right) with additional acetyl group highlighted in red

Previous studies have identified *N*-acetyl neuraminic acid (sialic acid, Neu5Ac) as both a potential biomarker for CVD such as heart failure, and risk of future cardiovascular events such as heart attack and stroke [5]. Neu5Ac concentrations were shown to be clearly elevated in plasma taken from patients with CVD *versus* plasma taken from healthy controls. Neu5Ac is a monosaccharide with a nine-carbon backbone that is generally located as the terminating unit of *N-* and *O*-glycans. In turn, these glycans form parts of glycoproteins and other glycoconjugates [6]. Neu5Ac is one of a family of over 50 sialic acids. The most abundant sialic acids present in humans are Neu5Ac and Neu5,9Ac_2_ [7] (Fig. 1), with Neu5,9Ac_2_ present in quantities around 100–200 times less than Neu5Ac. Neu5Ac is ubiquitous in the body, while Neu5,9Ac_2_ has been identified in biological fluids (blood, urine, saliva), in the brain, lungs, kidneys, intestines, and pancreas [8, 9].

Neu5Ac has many functions, primarily as a receptor mask or determinant which aids in cell-cell recognition and immune response, generally through the mechanism of receptor binding [10]. The carboxylic acid functional group conveys an overall negative charge to the cell surface and the endothelium which aids in cell repulsion [6] and the prevention of cell aggregation, especially among erythrocytes [11]. Neu5Ac and Neu5,9Ac_2_ expression on the surface of glycoproteins improves their circulating half-life in the blood [12]. These sialic acids have also been shown to have an anti-inflammatory effect. This takes place through various mechanisms such as the reduction of recruitment of leukocytes and the suppression of specific immunogenic proteins such as interleukins [13, 14]. This can help to protect cells from damage during an inflammatory state but can also be utilised by cancer cells to protect against the immune system [15–17]. Further to this, inflammation is associated with the acute-phase response, with a marked increase or decrease in specific acute-phase proteins during inflammation [18]. Acute-phase proteins are generally highly sialylated and as such would contribute to increased sialylation levels in the blood during an inflammatory state, such as that associated with CVD [19]. Neu5,9Ac_2_ concentrations may also appear upregulated due to a potential reduction in plasma esterase activity during an inflammatory state. Hubbard et al. reported significant associations between elevated inflammatory markers hs-CRP, interleukin-6 (IL-6), tumour necrosis factor alpha (TNF-alpha), and reduced plasma esterase activity [20]. This reduced plasma esterase activity may reduce the conversion of Neu5,9Ac_2_ to Neu5Ac hence resulting in elevated concentrations of Neu5,9Ac_2_ during an inflammatory state.

Previous research in this area has utilised assays which suffer from poor specificity for sialic acid which can lead to inaccurate results [21]. Quantitative analysis of these sialic acid derivatives as biomarkers for CVD requires a sensitive and specific analytical technique with a sufficiently low limit of detection. This is because Neu5Ac is present in sufficiently large quantities (average 45.49 mg/100 mL in healthy controls), with Neu5,9Ac_2_ being present in quantities 100–200 times less (average 0.29 mg/100 mL in healthy controls) [22]. Ideally, the assay of choice will also analyse total sialic acid and not just sialic acids present on *N*-glycans. Labelling of sialic acids with 1,2-diamino-4,5-methylenedioxybenzene dihydrochloride (DMB) followed by ultra-high performance liquid chromatography (UHPLC) analysis was chosen in this instance as it allows for the analysis of multiple types of sialic acid from all sources found in plasma in the same assay while exhibiting high specificity for them and a low limit of detection (< 0.01 nmol) and quantitation (< 0.04 nmol) [23, 24]. Like all quantitative techniques, the DMB assay requires quantitative standards for effective and accurate quantitation of sialic acids. Neu5Ac is commercially available in sufficiently large quantities but Neu5,9Ac_2_ is not, and therefore must be chemically synthesised. For the accurate quantitation of Neu5,9Ac_2_ in plasma samples, we have previously synthesised Neu5,9Ac_2_ and analysed the standard using quantitative nuclear magnetic resonance (QNMR) techniques [22].

A small-scale pilot study was designed herein to measure Neu5Ac and Neu5,9Ac_2_ concentrations in plasma samples from both healthy controls (n = 30) and CVD cohorts (n = 30). Analysis was carried out to determine if the elevation of concentrations of Neu5Ac and Neu5,9Ac_2_ between the healthy and disease cohorts was statistically significant. ROC curves were prepared to determine the sensitivity and specificity of each marker (Neu5Ac, Neu5,9Ac_2_ and a combined marker of Neu5Ac + Neu5,9Ac_2_) for the prediction of CVD. The same analysis was carried out for hs-CRP, which was measured in all samples, to allow for a comparison to a more well-established marker of inflammation and CVD. Hs-CRP was also combined with each of the sialic acids biomarkers as well as the combined Neu5Ac + Neu5,9Ac_2_ marker to determine any affect on the predictive power.

## Materials and methods

### Study population

30 Plasma samples from patients (16 female; 14 male) with an average age of 65 ± 22 with CVD were selected and purchased from the BioIVT biobank along with 30 age and sex matched healthy controls with an average age of 60 ± 13. Samples were chosen from volunteers that had one or multiple diagnosed CVDs including: hypertension, hypercholesterolemia, atrial fibrillation, congestive heart failure, coronary artery disease, but no other health conditions that could otherwise affect plasma sialic acid concentration such as type-2 diabetes [25], arthritis [26] or chronic obstructive pulmonary disorder (COPD) [27]. These conditions were chosen based on a literature search for health conditions associated with elevated plasma concentrations of sialic acid. Full details of the cohort can be found in Appendix 1 of the supplementary information.

### Analytical methods

Analysis of sialic acids was carried out using the DMB method [22, 24, 28]. Two samples were required per analysis, one to quantify Neu5Ac and one to quantify Neu5,9Ac_2_. Release of sialic acids and DMB labelling of the samples was achieved using LudgerTagTM DMB Sialic Acid (LT-KDMB-96). 5 μL of each sample was added in triplicate to a 96-well plate. Each sample was subjected to acid release with 25 μL of 2 M acetic acid. The samples were vortexed and centrifuged (RCF 1677) followed by incubation at 80^o^C for 2 h. The samples were cooled to room temperature before 5 μL of each released sample was transferred to a new 96-well plate. To this, 20 μL DMB labelling solution was added. The samples were vortexed and briefly centrifuged (RCF 1677) followed by incubation for 3 h at 50^o^C. The reaction was quenched by addition of water to make-up the volume to 500 μL. Neu5Ac samples were then subjected to a 1 in 10 dilution, Neu5,9Ac_2_ samples were not. All work was carried out using a Hamilton STARlet Liquid Handling Robot, apart from the initial dispensing of the samples into the 96-well plate.

Fetuin derived from fetal calf serum (GCP-Fet-50U), an A2G2S2 [29] glycopeptide (BQ-GPEP-A2G2S2-10U) both from Ludger Ltd. and Visucon-F frozen control plasma from Affinity Biologics were utilised as system suitability standards. These standards were subjected to the same release and labelling conditions as stated above for the samples containing Neu5Ac.

**Scheme 1:**
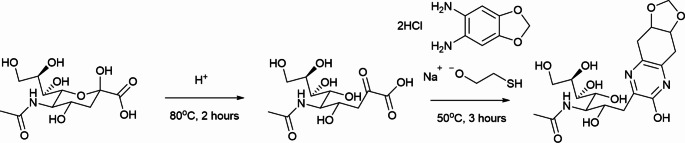
Sialic Acid release and DMB labelling reaction

One nmol standards of Neu5Ac and Neu5,9Ac_2_ [22] were also labelled using DMB (Scheme 1). 20 μL of labelling solution (3.5 mg DMB dye, 2.2 mL mercaptoethanol (1.4 M), 20 mg sodium dithionite) was added to each standard. The samples were vortexed and centrifuged (RCF 1677) before incubation for 3 h at 50^o^C. The labelling reaction was quenched with water to bring the final volume to 500 μL. Standard curves were prepared for each standard with points: 0.01, 0.02, 0.1, 0.2, 0.5, and 1.0 nmol.

The labelled sialic acids were analysed by LC-FLD. 5 μL of sample was injected into a U3000 UHPLC equipped with a fluorescence detector (λ_ex_ = 373 nm, λ_em_ = 448 nm, Thermo, UK) and separated on a C18 LudgerSepUR2 column (10 cm x 2.1 mm, 1.9 μm particles) at 30 ^o^C. For Neu5Ac analysis an isocratic solvent system (7:9:84 MeOH:ACN:H_2_O) was used. For Neu5,9Ac_2_ analysis a gradient solvent system was used of 7:6:87 MeOH:ACN:H_2_O for 6.5 min followed by 6:9:85 MeOH:ACN:H_2_O for 11.5 min.

Analysis of hs-CRP was carried out using an Invitrogen CRP human ELISA kit purchased from Thermofisher. Each sample was analysed according to the manufacturer instructions.

### Statistical analysis

Data are presented as mean ± standard deviation. p = 0.05 was used as the threshold for statistical significance. Differences in mean values were estimated using a two-sided t-test. Analysis was performed using R version 4.1.1 (RRID: SCR_001905) [30]. ROC curves were prepared using a Support Vector Classifier model (RRID: SCR_019053) [31] using the individual traits (Neu5Ac and Neu5,9Ac_2_ concentrations) as predictors, followed by a combination of both. The model was trained on 70% of the data and tested on the remaining 30% after data was standardised such that it followed a normal distribution. The model performance was evaluated by a five-fold cross validation. Predictive power was evaluated using AUC, information on sensitivity and specificity was also obtained. No outliers were identified. The outcomes were whether a given person had a diagnosed CVD (CVD cohort) or whether they had no diagnosed CVD (control cohort).

## Results

**Fig. 2:**
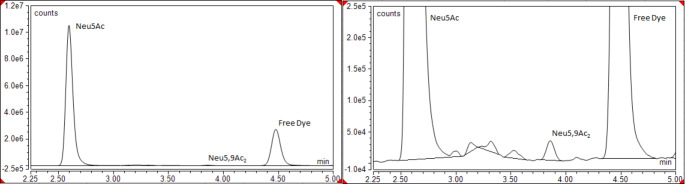
LC traces showing Neu5Ac and Neu5,9Ac_2_. Left: Regular view; Right: Zoomed view to highlight Neu5,9Ac_2_

In this study, the suitability of both Neu5Ac and Neu5,9Ac_2_ as novel diagnostic markers of CVD was investigated. The DMB assay employed for the analysis of Neu5Ac and Neu5,9Ac_2_ exhibited low levels of inter and intra-assay variation (< 10%). Neu5Ac and Neu5,9Ac_2_ were detected in each sample (Fig. 2). The concentrations in all samples met the criteria to overcome the limit of detection and limit of quantitation (three times and nine times signal to noise ratio respectively).

**Fig. 3:**
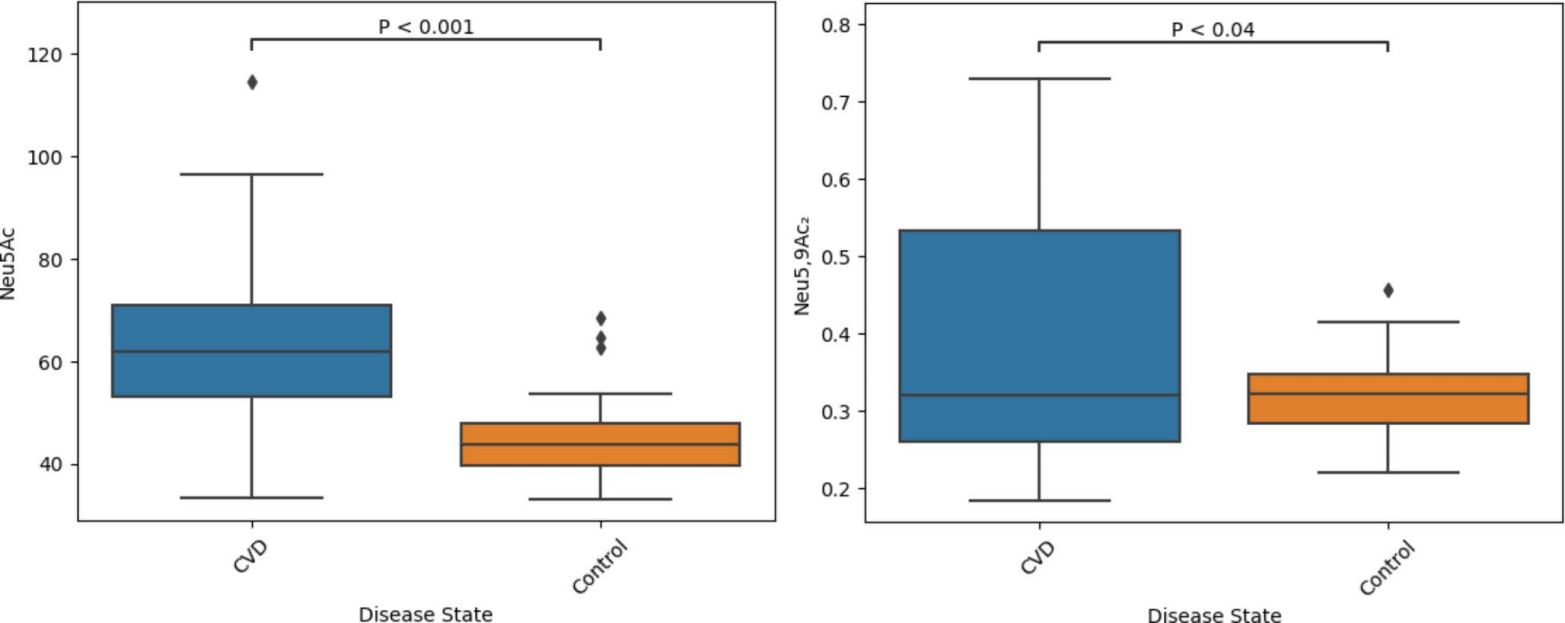
Boxplots showing the difference in plasma concentrations of Neu5Ac and Neu5,9Ac_2_ between CVD cases and healthy controls

The cohort characteristics are shown in Table 1. The statistically significant elevation of Neu5Ac between healthy controls and disease patient cohorts (45.19 ± 8.46 *versus* 63.55 ± 17.49; P < 0.001) (Fig. 3) in this study is supported by, and aids in confirming, the findings of previous work. The presence of atherosclerosis, hypertension, coronary heart disease and heart failure have all previously been associated with increased Neu5Ac concentrations [5]. Neu5,9Ac_2_ has not been previously investigated as a marker in the context of CVD. A statistically significant elevation in Neu5,9Ac_2_ concentration between healthy controls and CVD is reported here (0.32 ± 0.06 *versus* 0.40 ± 0.19; P < 0.04) (Fig. 3).

**Table 1:** Cohort characteristics. Data are shown as mean ± SD

	Healthy Controls	CVD Patients
n	30	30
Age (years)	65 ± 13	59 ± 22
Male:Female ratio	14:16	14:16
Mean Plasma Neu5Ac (mg/100 mL)	45.19 ± 8.46	63.55 ± 17.49
Mean Plasma Neu5,9Ac_2_ (mg/100 mL)	0.32 ± 0.06	0.40 ± 0.19
Mean Plasma hs-CRP (mg/ L)	1.85 ± 2.37	6.21 ± 15.25

**Fig. 4:**
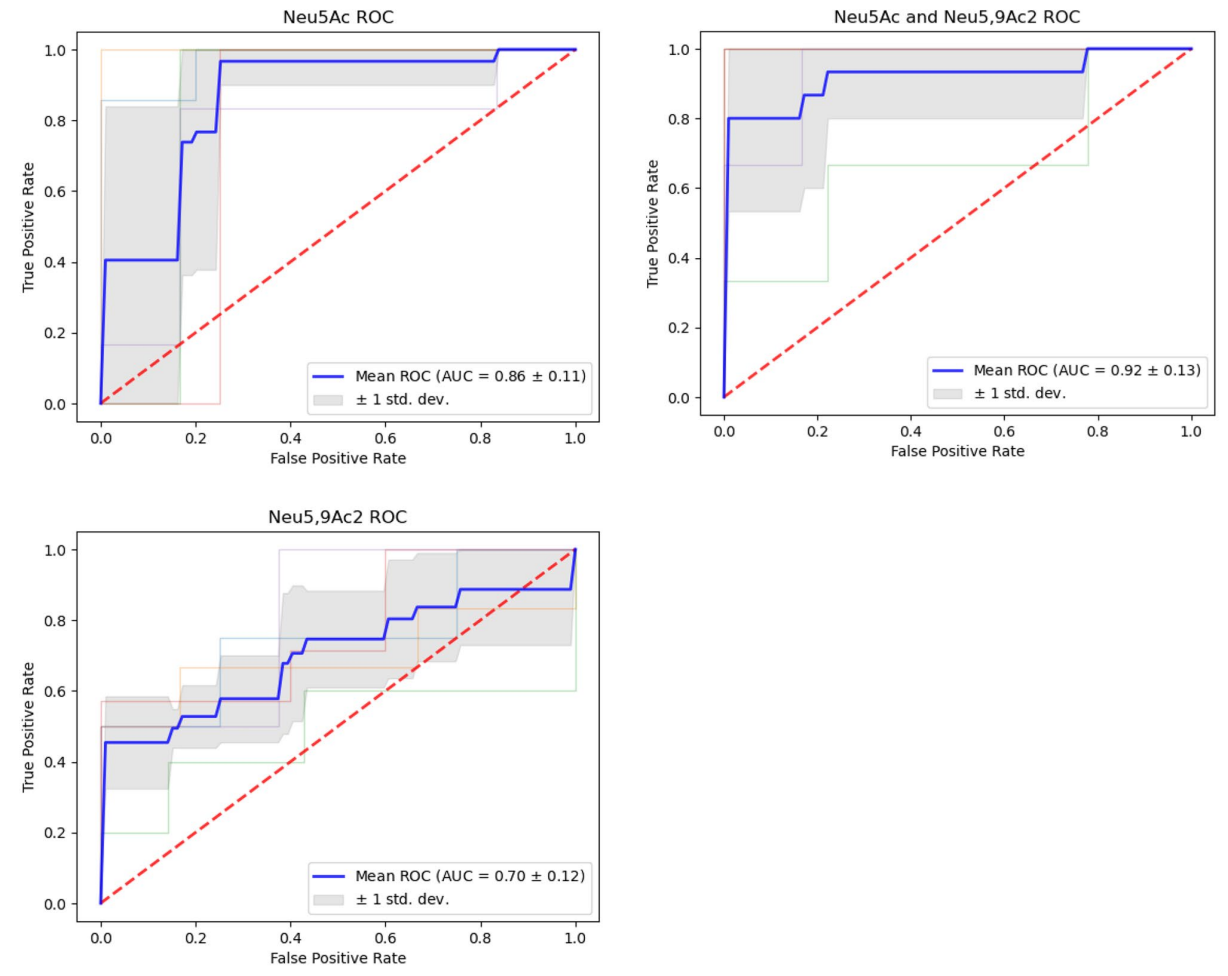
ROC curves for Neu5Ac, Neu5,9Ac_2_ and Neu5Ac + Neu5,9Ac_2_

ROC analysis showed that plasma Neu5Ac and Neu5,9Ac_2_ concentrations can be used to discriminate between CVD cases and healthy controls and thus their potential utility as biomarkers has been highlighted. The AUC, or ability to distinguish between the CVD cases and healthy controls, was calculated for plasma concentrations of Neu5Ac (0.86), Neu5,9Ac_2_ (0.71) as well as Neu5Ac + Neu5,9Ac_2_ as a combined marker (0.93) (Fig. 4). Neu5Ac was shown to have good predictive power for the CVD cases *versus* healthy controls. Neu5,9Ac_2_ performed less well in this regard with a lower AUC value. The improved predictive power of Neu5Ac over Neu5,9Ac_2_ indicated that it may be a better predictor of CVD. Interestingly, the combined marker for Neu5Ac + Neu5,9Ac_2_ showed a marked improvement in terms of AUC when compared to either of the individual markers. This appears to indicate that while Neu5,9Ac_2_ may not have high utility as a marker by itself, it offers an improvement on a previously utilised biomarker for CVD. Neu5Ac exhibited high specificity (0.81) and sensitivity (0.82), Neu5,9Ac_2_ exhibited equivalent specificity (0.82) but very low sensitivity (0.44). This data shows that both markers have a low false positive rate, however Neu5,9Ac_2_ poorly discriminates the positive results (CVD cases) and as such would not be a good marker in this context. Neu5Ac, however, performs very well in this cohort in all aspects. The combined marker Neu5Ac + Neu5,9Ac_2_ is interesting in that it offers both higher sensitivity (0.87) than that of Neu5Ac, but also higher specificity (0.90). This data is summarised in Table 2. Comparing the three markers overall shows that Neu5Ac is a good potential biomarker for CVD which backs up previous research carried out in this area [5]. Neu5,9Ac_2_ however did not perform as well in the capacity as a biomarker. This could be due to the low concentrations of Neu5,9Ac_2_ in plasma compared to Neu5Ac. Neu5,9Ac_2_ did offer some benefit, however, in that when combined with Neu5Ac, it was found that this combined biomarker offered a good improvement in terms of AUC, sensitivity and specificity over the previously established marker of Neu5Ac.

**Table 2:** ROC analysis data. Data are shown as mean ± SD

Marker	Sensitivity	Specificity	F-Score	AUC
Neu5Ac	0.82 ± 0.16	0.81 ± 0.17	0.80 ± 0.12	0.86 ± 0.12
Neu5,9Ac_2_	0.44 ± 0.22	0.82 ± 0.17	0.51 ± 0.20	0.71 ± 0.12
Neu5Ac + Neu5,9Ac_2_	0.87 ± 0.13	0.90 ± 0.12	0.88 ± 0.13	0.93 ± 0.10
hs-CRP	0.70 ± 0.41	0.31 ± 0.43	0.17 ± 0.24	0.50 ± 0.14

**Fig. 5:**
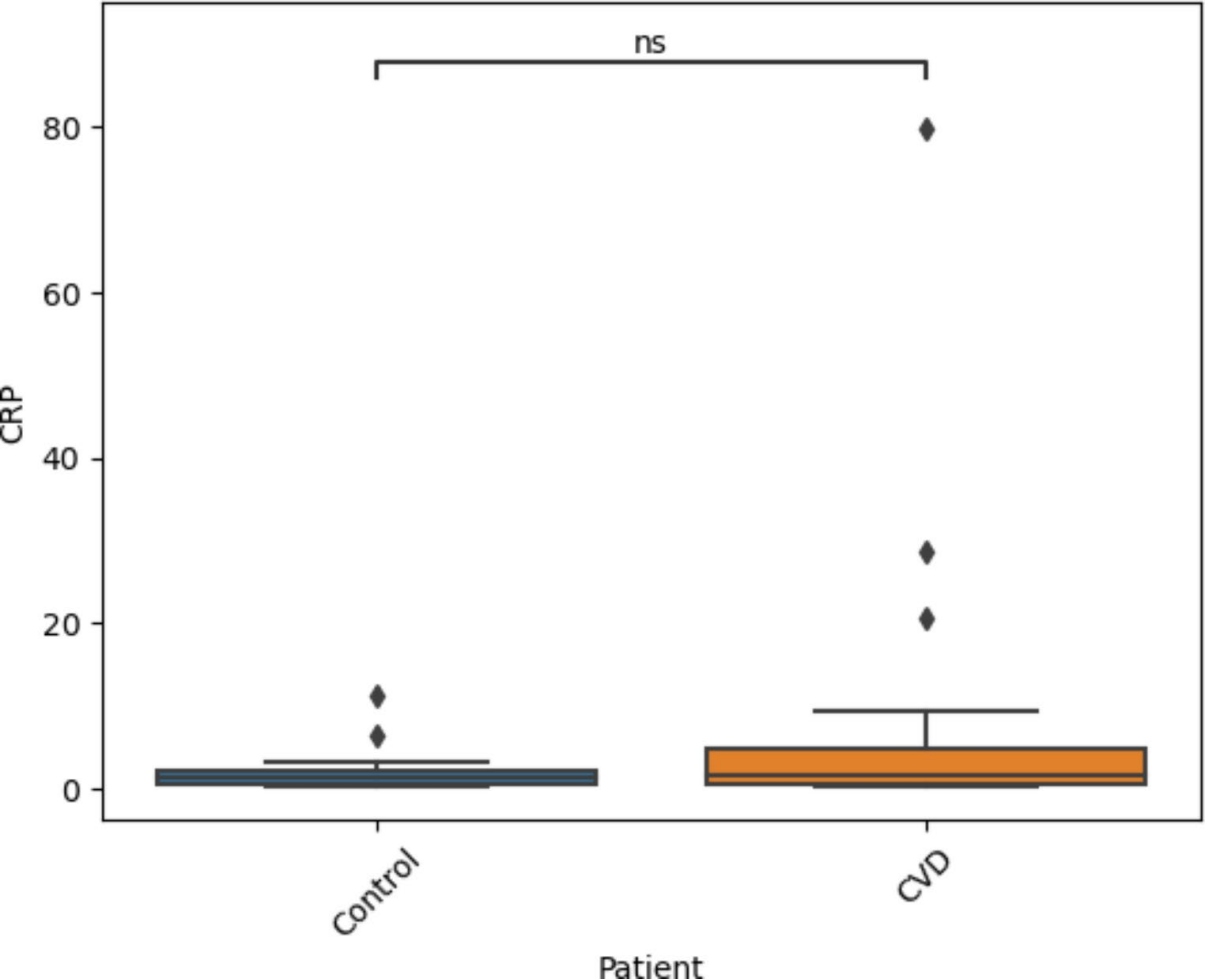
Boxplots showing the difference in plasma concentrations of hs-CRP between CVD cases and healthy controls

Further to this, hs-CRP was analysed in both the CVD and healthy control cohorts. This was performed to allow for a direct comparison of the markers detailed in this work against a well-established inflammatory marker. The mean values for each cohort were determined (6.21 ± 15.25 *versus* 1.85 ± 2.37) (Table 2) and the dataset was subjected to the same statistical analysis as the sialic acid biomarkers discussed above. The mean hs-CRP values were not significantly different between the CVD patients and healthy controls (Fig. 5).

**Fig. 6:**
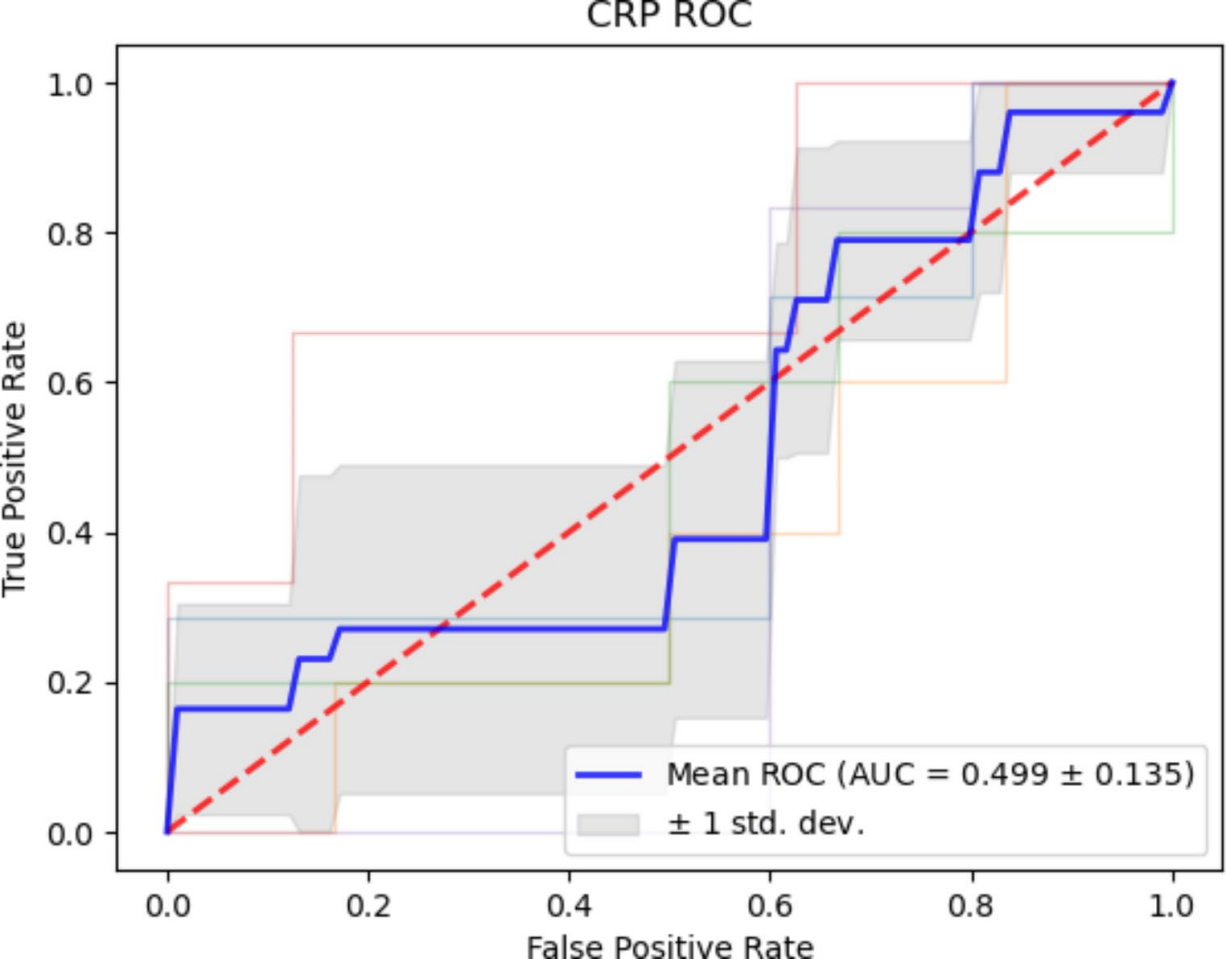
ROC curve for hs-CRP

ROC analysis was performed to determine what predictive power, if any, hs-CRP had for discriminating between CVD cases and healthy controls in this cohort. The AUC obtained was 0.50 ± 0.14 which is a very poor result, showing no ability for hs-CRP to be utilised for the discrimination of CVD cases from healthy controls beyond random chance for this particular cohort. Comparing this more well-established marker to the markers outlined in this paper highlights the potential utility of Neu5Ac and Neu5,9Ac_2_ as biomarkers. This is especially highlighted when comparing the AUC of hs-CRP to that of the combination marker Neu5Ac/Neu5,9Ac_2_ (0.50 ± 0.14 versus 0.93 ± 0.10) (Fig. 6).

Table 3 details the AUC values obtained when combining the hs-CRP with each of the sialic acid markers and the combined biomarker. Interestingly, despite the poor performance of hs-CRP as an individual marker for CVD, it increased the AUC when combined with the individual sialic acids and the combined marker. The combined biomarker reaching an AUC of 0.97 ± 0.05 with very high sensitivity and specificity.

**Table 3:** ROC analysis data. Data are shown as mean ± SD

Marker	Sensitivity	Specificity	F-Score	AUC
Neu5Ac + hs-CRP	0.75 ± 0.17	0.89 ± 0.15	0.80 ± 0.13	0.92 ± 0.10
Neu5,9Ac_2_ + hs-CRP	0.62 ± 0.21	0.80 ± 0.18	0.66 ± 0.16	0.76 ± 0.06
Neu5Ac + Neu5,9Ac_2_ + hs-CRP	0.90 ± 0.11	0.95 ± 0.10	0.92 ± 0.08	0.97 ± 0.05

## Discussion

Inflammation is a component of CVD that can result in myocardial and endothelial damage [32, 33]. An increase in inflammation related to CVD can lead to an acute-phase response which is characterised by upregulation of specific proteins such as alpha-1-acid glycoprotein, alpha-1-antitrypsin, fibrinogen, alpha-2-macroglobulin and hemopexin [34]. A variety of sialylated glycans decorate the surface of these proteins. A2G2S2 is the main glycan present in human plasma and is present in large quantities on alpha-1-antitrypsin and hemopexin. Alpha-1-acid glycoprotein has been reported to account for nearly all highly sialylated *N*-glycan species in plasma circulation [19]. These glycoproteins contain large quantities of sialic acids and an increase in the concentrations of these proteins during an acute-phase response would account somewhat for elevated Neu5Ac and Neu5,9Ac_2_ concentrations in plasma. Further to this, elevated sialic acid concentrations might mean that the sialic acid can act as a substrate for the resialylation of low-density lipoprotein (LDL) and erythrocytes. Desialylated LDL and erythroctyes have been found to aggregate more than unmodified variants thus leading to the build-up of atherosclerotic plaques [35, 36]. This is perhaps supported by evidence of an increase in the activity of sialyltransferase during an inflammatory state, which is perhaps an attempt to resialylate these structures and prevent cardiovascular damage occurring [37]. On the other hand, downregulation of the activity of plasma esterases has been observed with increased levels of inflammation [20]. This may reduce the quantity of acetylated sialic acid derivatives in plasma that are cleared by the activity of these enzymes, leading to the observation of higher than usual concentrations of these derivatives during an inflammatory state.

Hs-CRP is an acute-phase protein, and, as such, an increase in the protein concentration in plasma samples from patients with higher levels of inflammation would be expected. Hs-CRP is generally present in very low levels in individuals with no inflammation but rises quickly during an inflammatory state [38]. While hs-CRP is used as a measure of the acute phase response by healthcare organisations, some authors reviewing data from large-scale studies have called into question the utility of hs-CRP due to high variation in concentrations and low AUC value when it comes to predictive power [39, 40]. However, hs-CRP does appear to show some utility as a marker when combined with sialic acid markers.

To conclude, Neu5Ac and a combined Neu5Ac + Neu5,9Ac_2_ biomarker may have potential as biomarkers for CVD, possibly with some utility beyond currently utilised biomarkers such as hs-CRP. The sialic acid markers may be improved by combining with hs-CRP despite the poor performance of hs-CRP as an individual marker. It would be worthwhile to perform a follow-up study with a larger cohort size where the samples are sourced from a more well-controlled setting. Ideally, this would be from a single or family of well-described clinical sites with good clinical oversight to ensure the robustness of the sample collection with access to metadata for each sample. This would allow for the findings outlined here to be challenged and potentially reinforced. Including samples from patients with co-morbidities, one of the most common being diabetes, would also be valuable to determine the utility of these biomarkers in a wider, more representative, clinical setting. This would be particularly valuable if the co-morbidities also affect concentrations of plasma sialic acids independently of CVD and allow for the determination of whether these biomarkers can be utilised in a wider range of patients.

### Strengths and limitations

This study showed strength in the higher diversity of biomarkers that were studied compared to similar studies investigating sialic acids in the context of CVD. The standards used were also of high purity offering excellent quantitation. This was further backed up by the utilisation of a highly specific and sensitive method allowing for the accurate quantitation of sialic acid species in plasma without concern of interferences. Extremely small quantities of material could be detected with this method, allowing for effective quantitation of Neu5,9Ac_2_ which has not previously been reported.

The study was limited by the sample size which was small and will have resulted in an underpowered study, potentially explaining the borderline significance (P < 0.04) of plasma Neu5,9Ac_2_ concentrations between CVD cases and healthy controls. This may also have been explained by the relatively low concentrations of Neu5,9Ac_2_ in human plasma and the associated difficulty with measurement of low concentrations of analytes related to signal-to-noise ratio. The AUC value for hs-CRP may have been affected by the small samples size also highlighting the need for a larger future study. This was only a pilot study, however, and as such a higher-powered future study with more samples would help to supplement this preliminary data. Further to this, samples were selected only from patients with one or more CVDs, but no other co-morbid health conditions. As such, this may limit the utility of the biomarker and it would be useful to test these biomarkers in patients with CVD and co-morbid conditions such as diabetes.

## Conclusions

Neu5Ac and Neu5,9Ac_2_ concentrations were determined in samples from 30 healthy controls and 30 patients with diagnosed CVD. Neu5,9Ac_2_ could be detected and quantified with high precision even when present in very small quantities compared to Neu5Ac. Statistically significant elevations of concentrations for both Neu5Ac and Neu5,9Ac_2_ were observed. Neu5Ac was shown to be a good marker for the discrimination of these CVD patients from the healthy controls, Neu5,9Ac_2_ however suffered from low sensitivity and as such poor prediction of CVD cases. Interestingly, despite the poor performance of Neu5,9Ac_2_ as an individual marker, a combination marker of Neu5Ac + Neu5,9Ac_2_ offered improved predictive power over Neu5Ac alone. The markers identified here offer an improvement, in terms of AUC value, over currently utilised biomarkers for CVD. Neu5Ac, as shown in previous research, could be used as a potential biomarker for CVD diagnosis. This research also indicates that the addition of Neu5,9Ac_2_ is valuable resulting in improved predictive power for CVD diagnosis and Neu5Ac Neu5,9Ac_2_ could act as markers for the presence of CVD with excellent predictive power. Comparison to hs-CRP also highlighted the potential utility of the markers shown in this paper, with hs-CRP offering very poor predictive power compared to Neu5Ac and Neu5,9Ac_2_. Hs-CRP may offer some utility when combined with the sialic acid markers, offering a slight increase to AUC values. Future studies will be of benefit to solidify the results obtained in this research and to determine the utility of Neu5Ac as a biomarker in patients with CVD and co-morbid conditions. The outcome of this would have a great impact on the utility of the marker in a clinical setting where patients often present with multiple co-morbidities.

### Electronic supplementary material

Below is the link to the electronic supplementary material.

Supplementary Material 1

## Data Availability

The metadata for the patient cohorts in this study can be found in the supplementary information.
