# Supplementary material for: Elevated concentrations of Neu5Ac and Neu5,9Ac2 in human plasma: potential biomarkers of cardiovascular disease
Source: Glycoconj J. 2023 Nov 22;40(6):645–54. doi: 10.1007/s10719-023-10138-3 (PMC10788320; doi:10.1007/s10719-023-10138-3)
Supplement: Supplementary file 1 — Supplementary Material 1 [file 10719_2023_10138_MOESM1_ESM.docx]

**Elevated concentrations of Neu5Ac and Neu5,9Ac_2 ­_in human plasma: Potential Biomarkers of Cardiovascular Disease**

Jack Cheeseman^a,b^, Concepcion Badia^b^, Georgia Elgood-Hunt^b^ Richard Gardner^b^, Duong N. Trinh^c,e^, Marco P. Monopoli^c^, Gunter Kuhnle*^d^_,_ Daniel I.R. Spencer^b^, Helen M.I. Osborn*^a^

*Corresponding author, h.m.i.osborn@reading.ac.uk

^a^ School of Pharmacy, University of Reading, Whiteknights, Reading, UK. RG6 6AD

^b^ Ludger Ltd, Culham Science Centre, Abingdon, UK. OX14 3EB

^c^ Department of Chemistry, Royal College of Surgeons in Ireland (RCSI), Dublin 2, D02 YN77, Ireland

^d^ Department of Food and Nutritional Sciences, University of Reading, Whiteknights, Reading, UK. RG6 6AH

^e^ Department of Pharmaceutics and Pharmaceutical Technology, University of Medicine and Pharmacy, Vietnam National Univeristy, Hanoi, Vietnam

Glyconjugates

**BioIVT CVD case samples**

| Sex | Age (Years) | Race | Diagnosis | Neu5Ac (mg/100 mL) | Neu5,9Ac_2_ (mg/100 mL) | CRP (mg/L) |
| --- | --- | --- | --- | --- | --- | --- |
| Male | 40 | Caucasian | Hypertension, Intestinal Obstruction, Vitamin D Deficiency, Anemia | 51.09 | 0.18 | 0.20 |
| Male | 49 | Caucasian | Hypercholesterolemia, Hypertension | 67.51 | 0.27 | 1.27 |
| Male | 49 | Caucasian | Hypertension | 57.93 | 0.20 | 1.40 |
| Male | 52 | Caucasian | Hypertension | 70.94 | 0.22 | 0.22 |
| Male | 57 | Caucasian | Atrial Fibrillation, Hypertension | 61.93 | 0.26 | 0.32 |
| Male | 61 | Caucasian | Bipolar Disorder, Hypertension | 61.72 | 0.23 | 5.75 |
| Male | 67 | Caucasian | Hypertension, Atrial Fibrillation | 63.46 | 0.21 | 1.03 |
| Male | 70 | Caucasian | Congestive Heart Failure | 34.80 | 0.30 | 0.14 |
| Male | 72 | Caucasian | Hypertension, Hyperlipidemia | 71.02 | 0.32 | 0.63 |
| Male | 73 | Caucasian | Congestive Heart Failure, Vertigo, Hypertension, Hyperlipidemia, Ventricular Tachycardia, Ischemic Cardiomyopathy | 49.71 | 0.66 | 0.41 |
| Male | 75 | Caucasian | Congestive Heart Failure, Hypertension, Anemia, Hyperlipidemia | 64.20 | 0.69 | 28.51 |
| Male | 77 | Caucasian | Congestive Heart Failure | 54.71 | 0.40 | 0.33 |
| Male | 80 | Caucasian | Congestive Heart Failure, Hypertension | 35.55 | 0.64 | 1.65 |
| Female | 41 | Caucasian | Hypertension | 76.32 | 0.30 | 2.83 |
| Female | 51 | Caucasian | Hypertension | 69.29 | 0.29 | 6.62 |
| Female | 51 | Caucasian | Hypertension, Hyperchlolesterolemia | 52.40 | 0.69 | 0.14 |
| Female | 52 | Caucasian | Cardiovascular Disease, Hypertension | 72.59 | 0.36 | 2.46 |
| Female | 55 | Caucasian | Hyperuricemia, Hypertension | 91.20 | 0.39 | 20.61 |
| Female | 56 | Caucasian | Hypercholesterolemia, Hypertension | 96.33 | 0.38 | 2.54 |
| Female | 58 | Caucasian | Hypertension, Hypertriglyceridemia, Vitamin D Deficiency | 61.74 | 0.24 | 0.90 |
| Female | 59 | Caucasian | Hypertension, Hypercholesterolemia | 52.48 | 0.73 | 0.39 |
| Female | 63 | Caucasian | Hypertension | 78.95 | 0.25 | 1.05 |
| Female | 69 | Caucasian | Congestive Heart Failure | 57.48 | 0.28 | 2.29 |
| Female | 72 | Caucasian | Congestive Heart Failure, Sleep Apnea, Fibromyalgia, Gastroesophageal Reflux Disease, Hypertension, Hyperlipidemia, Aortic Stenosis, Paroxysmal Atrial Fibrillation, Pulmonary Hypertension | 51.13 | 0.71 | 5.27 |
| Female | 75 | Caucasian | Coronary Artery Disease, Hypertension | 76.21 | 0.32 | 0.45 |
| Female | 83 | Caucasian | Hypercholesterolemia, Hypertension, Hyponatremia, Pneumonia | 114.66 | 0.39 | 79.90 |
| Female | 84 | Caucasian | Congestive Heart Failure | 54.34 | 0.37 | 9.35 |
| Female | 87 | Caucasian | Hypertension | 68.37 | 0.28 | 6.45 |
| Female | 88 | Caucasian | Congestive Heart Failure, Atrial Fibrillation, Pulmonary Hypertension, Cardiovascular Disease | 55.18 | 0.71 | 1.48 |
| Female | 91 | Caucasian | Congestive Heart Failure, Atrial Fibrillation, Gastroesophageal Reflux Disease, Hyperlipidemia, Insomnia, Cerebrovascular Attack, Coronary Artery Disease | 33.18 | 0.58 | 1.54 |

**BioIVT Healthy Control Sample**

| Sex | Age | Race | Neu5Ac (mg/100 mL) | Neu5,9Ac_2_ (mg/100 mL) | CRP (mg/L) |
| --- | --- | --- | --- | --- | --- |
| Male | 45 | Caucasian | 41.19 | 0.32 | 0.18 |
| Male | 69 | Caucasian | 34.14 | 0.26 | N/A* |
| Male | 72 | Caucasian | 48.45 | 0.33 | N/A* |
| Male | 62 | Caucasian | 44.52 | 0.31 | 2.15 |
| Male | 40 | Caucasian | 49.54 | 0.23 | 0.32 |
| Male | 57 | Caucasian | 62.81 | 0.36 | 11.33 |
| Male | 62 | Caucasian | 43.93 | 0.23 | 0.53 |
| Male | 61 | Caucasian | 64.71 | 0.30 | 0.56 |
| Male | 64 | Caucasian | 45.15 | 0.35 | 1.57 |
| Male | 60 | Caucasian | 44.10 | 0.33 | 1.43 |
| Male | 65 | Caucasian | 68.37 | 0.32 | N/A* |
| Male | 55 | Caucasian | 46.33 | 0.22 | N/A* |
| Male | 46 | Caucasian | 51.86 | 0.32 | 2.58 |
| Female | 63 | Caucasian | 51.12 | 0.46 | 2.26 |
| Female | 48 | Caucasian | 43.23 | 0.33 | 1.10 |
| Female | 61 | Caucasian | 39.46 | 0.27 | 3.21 |
| Female | 59 | Caucasian | 39.14 | 0.38 | 0.75 |
| Female | 68 | Caucasian | 42.04 | 0.39 | 2.65 |
| Female | 61 | Caucasian | 38.41 | 0.35 | 0.63 |
| Female | 56 | Caucasian | 46.36 | 0.34 | 2.82 |
| Female | 56 | Caucasian | 40.41 | 0.39 | 6.32 |
| Female | 70 | Caucasian | 40.56 | 0.29 | 2.04 |
| Female | 59 | Caucasian | 39.01 | 0.28 | 0.21 |
| Female | 64 | Caucasian | 43.68 | 0.32 | 0.14 |
| Female | 64 | Caucasian | 35.80 | 0.36 | 2.03 |
| Female | 71 | Caucasian | 43.91 | 0.42 | 0.46 |
| Female | 63 | Caucasian | 43.41 | 0.31 | 0.66 |
| Female | 55 | Caucasian | 53.72 | 0.34 | 1.85 |
| Female | 62 | Caucasian | 37.27 | 0.25 | 0.16 |
| Female | 58 | Caucasian | 32.93 | 0.234 | 0.23 |

*Not available due to lack of available material for analysis
